# First Complete Genome Sequence of Barley Virus G Identified from Proso Millet (*Panicum miliaceum*) in South Korea

**DOI:** 10.1128/genomeA.00523-17

**Published:** 2017-07-20

**Authors:** Chung-Youl Park, Hyun-Geun Min, Jonghee Oh, Bong-Sub Kim, Seungmo Lim, Youngnam Yoon, Su-Heon Lee

**Affiliations:** a School of Applied Biosciences, Kyungpook National University, Daegu, South Korea; b Crop Production Technology Research Division, NICS, RDA, Miryang, South Korea; c Institute of Plant Medicine, Kyungpook National University, Daegu, South Korea

## Abstract

The complete genome sequence of an Uiseong isolate of barley virus G (BVG) on proso millet plants in a field in South Korea was determined by RNA sequencing and Sanger sequencing. To our knowledge, this is the first complete genome sequence report of BVG infecting proso millet in South Korea.

## GENOME ANNOUNCEMENT

Proso millet (*Panicum miliaceum*; Korean name, gijang) is a valuable cereal crop mainly consumed by humans in South Korea and Africa (1). Additionally, it is consumed as a breakfast powder and had been used for animal feed (2, 3). To date, research has mainly focused on its health benefits, such as its nutrient composition (4, 5). In recent years, proso millet demand has increased gradually, as the demand for potential functional foods in general has increased, but it has not been studied pathologically to evaluate viral disease. Moreover, only one virus (*Rice stripe virus* [RSV]) has been reported in South Korea (6).

To investigate the viruses infecting proso millet, during the growing seasons of 2015 and 2016, a total of 81 proso millet plants showing virus-like symptoms were collected in major cultivation region (6 provinces and 9 counties) in South Korea. To identify viral diseases from collected samples, all samples were pooled, and total RNA was extracted using a commercial total RNA extraction kit (iNtRON, Daejeon, South Korea) and analyzed by RNA sequencing. The total RNA quality check, rRNA removal, and library construction were performed for the Illumina HiSeq 2500 sequencing (Illumina, San Diego, CA, USA), as described previously (7). The raw data processing, *de novo* assembly, ordering of contigs, and annotation were performed by the SEEDERS Company (Daejeon, South Korea). A single contig of 5,580 nucleotides (nt) shared a sequence identity of 98% with barley virus G (BVG), with 100% query coverage. To determine the complete genome sequence of the BVG Uiseong isolate, 7 primer pairs were designed based on the contig sequence, and rapid amplification of cDNA ends was performed. All PCR products were cloned and sequenced by Sanger sequencing. The BVG Uiseong isolate was 5,620 nt in length (accession no. LC259081), including 6 open reading frames, consistent with a previously reported BVG Gimje isolate (accession no. KT962089). The 5′ and 3′ untranslated regions (UTRs) were 53 nt and 139 nt, respectively. The complete genome of the BVG Uiseong isolate had the highest nucleotide sequence identity (98%) with the BVG Gimje isolate. BVG belongs to the tentative genus *Polerovirus* in the family *Luteoviridae*; the virus was first reported on barley in South Korea (8). To determine the infection rate of BVG, total RNA was extracted from all 81 collected samples (2015, *n* = 60; 2016, *n* = 21) and tested by reverse transcription-PCR (RT-PCR) with BVG-specific primers (9). The RT-PCR results showed that 17 samples (2015, *n* = 7; 2016, *n* = 10) were positive for BVG. During the 2-year sampling period, the BVG infection rate increased. These results imply that the presence of BVG could pose a potential threat to Gramineae plants.

To date, only one complete genome sequence of BVG has been deposited in the NCBI GenBank database. In this work, we determined the complete genome sequence, for the first time to our knowledge, of BVG isolated from proso millet in the world.

### Accession number(s).

The nucleotide sequences of BVG Uiseong isolate have been deposited in NCBI GenBank under the accession number LC259081.
